# The Effect of Exercise on Glucoregulatory Hormones: A Countermeasure to Human Aging: Insights from a Comprehensive Review of the Literature

**DOI:** 10.3390/ijerph16101709

**Published:** 2019-05-15

**Authors:** Maha Sellami, Nicola Luigi Bragazzi, Maamer Slimani, Lawrence Hayes, Georges Jabbour, Andrea De Giorgio, Benoit Dugué

**Affiliations:** 1 Sport Science Program, College of Arts and Sciences (QU-CAS), University of Qatar, Doha 2713, Qatar; msellami@qu.edu.qa (M.S.); gjabbour@qu.edu.qa (G.J.); 2 Postgraduate School of Public Health, Department of Health Sciences (DISSAL), University of Genoa, 16132 Genoa, Italy; maamer2011@hotmail.fr; 3 Active Ageing Research Group, Department of Medical and Sport Sciences, University of Cumbria, Bowerham Road, Lancaster LA1 3JD, UK; lawrence.hayes@cumbria.ac.uk; 4 Department of Psychology, eCampus University, 22060 Novedrate, Italy; andrea.degiorgio@uniecampus.it; 5 Laboratory of Mobility, Aging and Exercise (MOVE), EA 6314 Poitiers, France; benoit.dugue@univ-poitiers.fr

**Keywords:** glucose, insulin, cortisol, growth hormone, physical activity, advanced age and senescence

## Abstract

Hormones are secreted in a circadian rhythm, but also follow larger-scale timetables, such as monthly (hormones of the menstrual cycle), seasonal (i.e., winter, summer), and, ultimately, lifespan-related patterns. Several contexts modulate their secretion, such as genetics, lifestyle, environment, diet, and exercise. They play significant roles in human physiology, influencing growth of muscle, bone, and regulating metabolism. Exercise training alters hormone secretion, depending on the frequency, duration, intensity, and mode of training which has an impact on the magnitude of the secretion. However, there remains ambiguity over the effects of exercise training on certain hormones such as glucoregulatory hormones in aging adults. With advancing age, there are many alterations with the endocrine system, which may ultimately alter human physiology. Some recent studies have reported an anti-aging effect of exercise training on the endocrine system and especially cortisol, growth hormone and insulin. As such, this review examines the effects of endurance, interval, resistance and combined training on hormones (i.e., at rest and after) exercise in older individuals. We summarize the influence of age on glucoregulatory hormones, the influence of exercise training, and where possible, examine masters’ athletes’ endocrinological profile.

## 1. Introduction

The aging process is accompanied by one or more changes in biological functions (affecting nervous system, cardiovascular and respiratory systems, or renal function, amongst others), often associated with an increasing susceptibility to co-morbidities and mortality [1,2].

According to the World Health Organization (WHO), three categories of population can be distinguished: “young old” (65–74 years old), “middle aged” (75–84) and the oldest (85+). Generally, aging leads to an overall loss of tissue vitality through a myriad of signaling mechanisms [3].

The anatomical and physiological changes associated with aging start several years before the appearance of external signs. Many of these alterations gradually manifest in the third decade and continue until death. These changes are also accompanied by a gradual decline in physical fitness and physical activity. This alteration of the cardiovascular and respiratory systems during the aging process can be mainly explained by a decline in maximum oxygen uptake (~10% per decade) starting from the age of 20 [4,5,6,7].

Advancing age is also associated with a decline in anaerobic performance, which can be mainly explained by changes involving the neuromuscular system and a major loss in type II fibers. Indeed, advanced age is accompanied by muscular wasting, a decrease in the rate of contraction, and maximum force.

According to Korhonen et al. [8], the first decline in muscle strength and mass occurs around the age of 30 and the loss is around 15% per decade from the age of 50 to 30% at the age of 70. Moreover, available scholarly literature suggests that starting from the 4th decade of life, both skeletal muscle mass and strength decline in a linear fashion and within the 8th decade of life, 50% of mass will be lost [9]. Since muscle mass amounts to 60% of body mass, its pathological changes can have deep consequences in the elderly.

One hypothesis for the reduction in physical performance and muscle weakness associated with age is an alteration of the endocrine system [10,11,12,13,14]. In particular, the glucoregulatory system that is characterized by important bio-molecules such as glucagon and insulin is critical to maintain the constancy of glucose in the internal milieu. While it is clear that exercise training improves fitness and physical capacity in older adults [15,16,17,18,19], whether exercise can improve the hormonal profiles of older adults remains contentious [20,21,22,23,24,25,26,27].

Therefore, this review will summarize the existing literature concerning the influence of age, and the effects of each mode of exercise (endurance, sprint, and resistance training) on relevant (basal) hormones belonging to the glucoregulatory system.

Where possible, we will provide evidence from masters athletes involving the influence of lifelong exercise on these hormones, but also report findings from interventional studies providing information on the training effect on these hormones.

## 2. Materials and Methods

The present review was designed as a comprehensive review of the literature. Search strategy adopted in the present review is summarized in Table 1.

## 3. Insulin, Aging and Physical Activity

Insulin plays a key role in glucose uptake by muscle, fat, and liver cells. Moreover, insulin inhibits both the liver glucose production and its secretion in blood.

Recent reports suggest that the insulin/insulin-like growth factor-1 (IGF-1) signaling pathways and molecular cascades have an important, evolutionarily conserved influence over rate of aging and, thus, longevity [28]. The most important effects of advancing age on this hormone are the increase of fasting insulin and decrease in insulin sensitivity [29,30].

Many studies examined the effect of different training modes, volumes and intensities on insulin levels in older adults. From the available investigations, it appears that short-term (two weeks) training was unable to reduce fasting insulin level in a group of 28 healthy middle-aged (40–55 years) sedentary men, as shown by Heiskanen and coauthors [31]. More in detail, a program of six supervised cycle ergometer training sessions, characterized either by high-intensity (n = 14; 4–6 × 30 s all-out cycling/4-min recovery) or continuous moderate-intensity (n = 14; 40–60 min at 60% peak O_2_ uptake) training did not affect fasting insulin concentration.

In contrast, Kirwan et al. [32] reported that nine months of endurance training reduced fasting insulin and improved insulin action. Seals and colleagues [33] (12 months of endurance training program), Kahn and coworkers [34] (six months of intensive endurance exercise program), Evans and coauthors [35] (10–12 months of endurance training program) reported similar results. Therefore, it appears that an intervention with a longer duration (e.g., from six up to 9–12 months) is required to observe significant changes in fasting insulin in older adults. On the other hand, some studies investigating the effects of 6/9-month training programs, such as the investigation by Goulet et al. [36], Dipietro and coworkers [37] or Ihalainen and collaborators [38] failed to report beneficial changes in insulin concentration.

The length of the training program seems to have an impact on insulin (in terms of levels or activity) depending on the age group in which the intervention is carried out. Herbert et al. [23] reported a moderate decrease in basal insulin following six weeks of high-intensity interval training (HIIT) in sedentary older males, suggesting that sprint training can reduce fasting insulin in older adults. Guezennec et al. [39,40] have investigated the impact of four months of weight lifting in athletes aged ~35 years old. After maximal sessions, the level of insulin did not change significantly.

Other studies examined the effect of resistance training in insulin sensitivity in elderly subjects and reported that strength training induced improvement in insulin-stimulated glucose uptake promoted by glucose transporter type 4 (GLUT-4) in elderly [41]. Further studies investigated the influence of 12 weeks of high resistance training (weight lifting program) in the elderly and observed decreased insulin response [42].

Furthermore, when comparing young and middle-aged men, Sellami et al. [43] investigated the impact of 13 weeks of combined sprint and strength training on insulin concentration in blood. They reported a significant decrease in fasting insulin in both groups. Interestingly, the effect of age that was evident at baseline was no longer present post-training, suggesting that combined sprint and strength training can prevent the negative effects of aging in trained men [43].

From a molecular standpoint, it seems that lifelong regular physical activity leads to epigenetic mechanisms in terms of global DNA methylation patterns positively impacting on skeletal muscles’ functioning in aged healthy individuals. One study has recently found that DNA methylation was statistically significantly lower in 714 promoters of genes involved in glycogen metabolism, glycolysis, oxidative stress resistance and muscle contraction, activity and myogenesis, whereas, methylation of introns, exons and CpG islands was apparently independent of physical activity practice [38]. Other cellular mechanisms that can explain how exercise can mitigate the mandatory age-related change in insulin levels include GLUT expression and translocation, skeletal muscle capillarization, improving insulin activity and sensitivity and favoring glucose uptake [42,44,45,46,47,48,49,50,51,52].

Even if short-term training cannot affect insulin levels, it seems to be sufficient in improving or at least preserving insulin secretion pattern and response to oral glucose load. Some studies have, indeed, shown that a single bout of high intensity intermittent exercise [53], a couple of bouts of exercise [54,55] or light/moderate-intensity physical activity [56,57,58,59,60] can be sufficient in preserving insulin activity and response to oral glucose tolerance test (OGTT).

In other studies, the physical activity level (trained versus untrained) was self-reported and assessed through the administration of questionnaires [61,62,63,64,65] or via quantitative measurements, such as accelerometer [59]. Some studies included in the present comprehensive review were high-quality randomized or pseudo-randomized studies [66,67,68].

Summarizing (Table 2), based on the available studies, it appears that aging is associated with an increase of insulin level; a major part of this increase can be counteracted by exercise training. Exercise is, indeed, a full mediator of the relationship between inactivity time sedentary behaviors and insulin resistance [69]. Exercise, especially long-term (i.e., 12–24 weeks and not less than 8–10 weeks) [70,71,72] endurance, resistance and multimodal/combined training [73,74] or short-term (i.e., bouts of six weeks of HIIT) [75,76,77] training program, can positively impact on insulin levels [78], even though existing scholarly findings are not so clear-cut and warrant further investigations.

## 4. IGF-1, Aging and Physical Activity

The IGF1 gene is situated on the long arm of chromosome 12. IGF-I is an endocrine and autocrine/paracrine growth factor expressed by multiple cell types. It plays a key role in the growth of cells, muscle, cartilage, bone, skin, and controls cell growth. The concentration of IGF-1 in blood peaks around adolescence and then declines after middle-age. This reduction in anabolic hormones has been termed the ’somatopause’, and is suggested as a mechanism for the process of aging.

Importantly, IGF-1 is implicated in skeletal and muscle function, which deteriorates with age. Eight weeks’ endurance training increased systemic IGF-1 in ~66-year-old males by ~19% [79].

However, other studies failed to observe any change in IGF-I following six months’ endurance training in ~67-year-old males. Herbert et al. [23] investigated the difference between endurance-trained master athletes (~60 years) and lifelong sedentary older adults (~62 years) and observed greater serum IGF-1 concentration in the trained compared to the sedentary subjects (~18.4 vs. ~13.1 μg/dL, respectively). Moreover, when exposing sedentary individuals to an endurance training program of 150 min/week, there was a small, non-significant increase in IGF-1 (~8% increase).

In addition, few studies explored the influence of sprint training on IGF-1 in older adults. Herbert et al. [23] observed that old (~62 years) sedentary subjects experienced a large increase in IGF-1 following 12 weeks’ preconditioning and HIIT (~13.1 to ~16.9 μg/dL). Although six weeks of preconditioning of 150 min/week accounted for 8% of the change in IGF-1, HIIT was responsible for a further 21% increase (28% greater than baseline). Findings from the same study suggest a trivial change in IGF-1 post-HIIT in age-matched master athletes. Therefore, post-HIIT, the sedentary individuals and master athletes had IGF-1 concentrations that were not significantly different.

Furthermore, when looking at the alteration of IGF-1 after resistance training in older adults, Parkhouse et al. [80] observed an increase in ~68 year old females’ circulating IGF-1. However, a recent investigation reported decreased systemic IGF-1 following 12 weeks’ resistance training in older adults (74 ± 6 years) with an increase in lean mass [81].

As such, Arnason et al. [81] hypothesized that IGF-I was redistributed from circulation into tissue during periods of anabolism. As a result of the ambiguity in the findings, the role of IGF-I in the adaptive process to exercise during middle and older age remains unclear. The majority of studies reported that resistance training can increase the concentration of IGF-1 in blood and increase muscle mass and function [82,83,84,85,86,87,88,89,90,91,92,93,94,95,96,97]. Yet, more longitudinal studies are needed to explore the influence of resistance training on IGF-1 in older adults, given the presence of discrepancies among the findings.

In a recent study, Sellami et al. [43] investigated the influence of age on somatotropic hormones. They observed that young males had greater serum IGF-1 concentration than middle-aged men. Moreover, Sellami et al. [43] reported that 13 weeks of combined sprint and resistance training increased circulating IGF-1 in middle-aged participants. Furthermore, the effect of age that was apparent at the study commencement was abrogated post-training, suggesting that exercise can counteract the effect of age on IGF-1 in middle-aged men.

Taken together (Table 3), these data suggest that HIIT, resistance and combined training may be a countermeasure to the age and lifestyle-related reduction in IGF-1, activating some gene pathways and protein cascades [98,99].

## 5. Growth Hormone, Aging and Physical Activity

Growth hormone (GH) secretion decreases with age, resulting in a downstream reduction in IGF-1 levels. This change, termed as the somatopause, is associated with loss of vitality, muscle mass, physical function, and an increased risk of frailty, cardiovascular disease, and adiposity, amongst others [100].

Veldhuis et al. [101] showed that GH secretion during puberty varied between 1–1.5 mg/day, while elderly people can produce only 50 µg/day. Several factors may be responsible for this decline, such as physical inactivity, poor nutrition, and subsequent changes in body composition. Moreover, Khan et al. [102] found that GH pulse decreased, and this decline was related to the alteration of hypothalamic and somatostatin hormones.

Moreover, GH has a beneficial neuroprotective effect [103] mainly due to the activation of anti-apoptotic pathway [104], this one particularly studied in literature. GH is also able to act on brain derived neurotrophic factor (BDNF) and neurotrophin-3 (NT3) [103] which, in turn, are particularly sensitive to physical activity.

Until now, there have been no studies that have explored the impact of endurance training on GH in older adults. Deuschle et al. [105] studied 11 elderly male marathon runners compared to 10 age-matched male sedentary people (controls), in order to study plasma concentration of GH, total and free IGF-I/II and IGF-binding protein-1, 2, and 3 and insulin. In particular, authors did not find any differences between runner vs controls, except for IGF-binding protein-1 and 2 increased in runners.

Moreover, Vanhelder al. [106] found similar results with a group of men aged 24–54 years who participated in resistance training for one year. The program was composed of two exercises (exercise protocol 1: vertical leg lifts at 85% of the subjects seven repetition maximum (SRM)/exercise protocol 2: vertical leg lifts with one third of the previously used load). The results showed that GH increased immediately after 5, 10, 25 min of exercise protocol 1. However, there was no significant increase after exercise protocol 2. These findings suggest that the frequency, duration of exercise play an important role in the regulation of GH secretion. Generally, the available studies showed that the frequency and intensity of resistance training are important factors in the regulation of GH secretion.

Recently, Sellami et al. [43] reported that younger adults had greater GH at rest and in response to sprint exercise than middle-aged participants. However, 13 weeks of combined sprint and resistance training abrogated this age effect and increased GH at rest and post-exercise in both young and middle-aged participants.

Summarizing (Table 4), very few studies have investigated the effect of physical activity and training on GH levels in elderly subjects [74,107,108], generally reporting negative findings. Further studies are needed to elucidate the mechanism of exercise on GH.

## 6. Glucagon, Aging and Physical Activity

Glucagon is a peptide hormone, belonging to the secretin family of hormones, produced and released by the alpha cells of the pancreas. Being the major catabolic hormone of the human body, it increases blood glucose and fatty acids concentration, differently from insulin [109].

Of note, no studies investigated the effect of physical activity on glucagon concentration in elderly subjects, with the exception of Hagberg and coworkers [82], who found no changes in trained older subjects, whereas untrained individuals reported increases in glucagon levels.

## 7. Cortisol, Aging and Physical Activity

Cortisol, the primary stress hormone, is a steroid belonging to the glucocorticoid family, produced and released by the zona fasciculata of the adrenal cortex. This hormone plays a key role in controlling blood glucose and metabolism in general. Studies exploring the impact of age on cortisol have shown that cortisol increases with human aging. Seaton [110] reported that there was an elevation of night time cortisol levels in elderly individuals and this increase could be caused by stressful factors such as insomnia. Our laboratory has demonstrated that middle-aged men have higher basal cortisol concentrations than young men [111].

There are only a few studies that have examined the effect of exercise training on cortisol in elderly subjects. Herbert et al. [23] investigated the difference between lifelong sedentary and endurance-trained master athletes and observed no difference in basal cortisol. Moreover, when exposing sedentary individuals to an endurance training program of 150 min/week, there was no alteration to basal cortisol. Similarly, De Souza Vale et al. [112] investigated the effect of three months of water aerobics training in elderly women and reported no alteration to basal cortisol. However, an increase in cortisol following six-week HIIT in master athletes has been observed, with a concomitant increase in peak power output [23].

In middle-aged men, we have previously observed no alteration to basal cortisol following combined sprint and resistance training, however the acute cortisol response to a supramaximal sprint was elevated post-training [111].

Regarding the aging-related changes in the effect of exercise training on cortisol level, Kraemer et al. [113] compared the level of cortisol in young and older men after heavy resistance training three times per week for 10 weeks. Results showed a decline in resting cortisol at three and 10 weeks in the older group. However, Häkkinen et al. [114] reported that elderly subjects and middle-aged subjects did not experience any change in cortisol after six months’ progressive resistance training. Similarly, Izquierdo et al. [115] investigated the effect of 16 weeks of progressive resistance training in older and middle-aged participants and observed no change in cortisol in the middle-aged group, but a decrease in the elderly group.

In summary, given the ambiguity of cortisol adaptation to resistance training, more research is required to determine the effect of training variables (duration, intensity, volume, frequency) and participant characteristics (age, training status, sex) on cortisol level.

## 8. Cathecolamines, Aging and Physical Activity

Catecholamine levels have been found to be different between young (20-years-old) and middle-aged men (40-years-old), with plasma noradrenaline concentrations being significantly lower (*p* < 0.05) in the young group when compared to the aged group. However, the precise neurobiological mechanisms leading to this difference in concentration levels are not very well-known and conflicting findings have been reported in the literature.

For instance, Hoeldtke et al. [116] showed that basal plasma noradrenaline concentration was greater in the elderly due to age-affected sympathetic nervous activity or sensitivity to sympatho-adrenal stimulation, without any difference in noradrenaline clearance. On the other hand, other authors found that clearance of noradrenaline tended to diminish with advancing age, which may contribute to the increased plasma concentrations observed.

Of note, no study has examined the effects of exercise training on catecholamines in older adults. In fact, the majority of studies investigated the impact of different types of training (sprint, endurance, resistance training) on catecholamine in young individuals [117,118,119,120,121,122,123]. Results were found to be at variance, and most of the time it was concluded that duration, intensity and type of training (aerobic and anaerobic) are the principal factors that induced alterations in catecholamine responses.

A notable exception was the investigation carried out by Poehlman and Danforth [71], who assessed the effects of 8 weeks of an endurance training program on norepinephrine kinetics in a sample of 19 older persons aged 64 ± 1.6 yr. Resting concentrations of norepinephrine were found to be increased by 24% after cycling exercise due to a 21% increase in norepinephrine appearance rate, whereas no change in norepinephrine clearance could be detected.

As such, due to the dearth of data and information, future studies are needed to determine the effect of different exercise training modes and moderator variables on catecholamine secretion and catecholamine circulating concentration in older adults.

## 9. Discussion and Future Prospects

There is an increasing interest in exercise training, as a therapeutic lifestyle strategy to attenuate the hallmarks of aging and improve health. Exercise training attenuates many markers of biological aging and one of the underlying mechanisms may be through the promotion of a more ‘youthful’ endocrine profile. In vitro experiments suggest that cells treated with plasma isolated from younger individuals are healthier or more ’youthful’ than those treated with plasma from their older peers. Therefore, in situ cells exposed to a youthful systemic environment will likely have improved functioning compared to those exposed to an older systemic environment. Evidence cited in this review suggests that it is possible that exercise can act as a countermeasure to endocrinological aging.

Regarding this last point, it is necessary to keep in mind that both similarities and differences in aging between/within genders exist.

However, despite such an increasing body of interest, the physiological effects of physical activity and exercise on glucoregulatory hormones in elderly subjects are relatively understudied. Evidence of the impact of training is generally circumstantial and randomized studies, carried out with high methodological rigor and quality are few or lacking for some hormones. Whereas insulin has captured the attention of scholars, there is a relative dearth of data and information for other hormones.

Given the importance of the topic of counter-aging effect of sports and physical activity and considering the epidemiological and clinical burden of aging and age-related disorders, more attention in the field is needed. Longitudinal studies employing larger sample sizes are warranted.

## Figures and Tables

**Table 1:** Search strategy adopted in the present comprehensive review of the literature for retrieving studies investigating the effects of physical activity and exercise on glucoregulatory hormones in elderly subjects.

Search Strategy Item	Details
Search string	(old OR elderly OR effect of age OR aging OR ageing) AND (physical activity OR sport OR exercise OR training) AND (insulin OR glucagon OR growth hormone OR IGF-1 OR glucoregulatory hormones OR cortisol OR catecholamines)
Searched databases	PubMed/MEDLINE, Scopus, ISI/Web of Science
Inclusion criteria	P (population): older subjects in good health
	I (intervention) / E (exposure): physical activity interventions; exposure to physical activity
	C (comparator / comparison): young subjects (both trained and untrained) and old untrained subjects
	O (outcome): changes in glucoregulatory hormones levels
	S (study design): original, primary research article
Exclusion criteria	P (population): young subjects; old frail subjects or with diseases (diabetes, obesity)
	I (intervention) / E (exposure): not exposed to physical activity / sports /exercise interventions or exposed to combined interventions (dietary intervention, supplementation, pharmacological treatment or other forms of manipulation) from which it was not possible to dissect the effect of training only
	C (comparator / comparison): absence of comparisons between age groups
	O (outcome): changes in glucoregulatory hormone levels not reported in detail or not clear
	S (study design): not original study (commentary, review, expert opinion, letter to editor, editorial)
Time filter	None applied (from inception)
Language filter	None applied (any language)

**Table 2:** Studies investigating the effects of physical activity and exercise on insulin in elderly subjects.

Authors	Study Year	Sample Size	Age	Gender	Intervention	Main Findings
Seals et al. [33]	1984	11	63 ± 1 y	Male and female	12 months of endurance training (low-versus high-intensity program)	Improved insulin sensitivity and reduction in total AUC for insulin by 8–23% (by 8% after the low-intensity training program and by 23% after the high-intensity training program)
Seals et al. [61]	1984	12	62 ± 1 y	Male	Self-reported physical activity	Lean older subjects had similar insulin levels when compared to younger subjects and statistically lower than the older untrained individuals
Hollenbeck et al. [62]	1985	20 (13 inactive versus 7 active subjects)	60–75 y	Male	Self-reported physical activity level	Better insulin resistance profile in older trained subjects
Craig et al. [42]	1989	9 (cases versus 6 young controls)	62.8 ± 0.7 y	Male	12 weeks of progressive high-resistance training (weight lifting program with a three set, six to eight repetition protocol: 45–60 min of isotonic weight-conditioning exercise on Nautilus equipment and leg press, leg extension, leg curl, torso extension, bench press, pull down, pull over and horizontal arm adduction)	Reduction in insulin levels
Tonino [70]	1989	11	60–80 y	Male	12 weeks of physical training	Decrease in peripheral insulin resistance
Kahn et al. [34]	1990	13	61–82 y	Male	6 months of intensive endurance exercise training	Decrease of insulin levels
						Increase of insulin sensitivity by 36%
Broughton et al. [63]	1991	13 (cases versus 14 young controls)	60 y and older	Male	Self-reported physical activity level	No significant differences
Poehlman and Danforth [71]	1991	19	64 ± 1.6 y	Male	8 weeks of endurance training program (cycling exercise)	No changes in insulin levels
Kirwan et al. [32]	1993	12	65 ± 1 y [60–70 y]	Male	9 months of endurance training	Reduction in fasting insulin
						Improved insulin activity
Cononie et al. [75]	1994	9	60–80 y	Male	Seven days of 50 min of exercise at 70% VO_2max_	Fasting plasma insulin levels and plasma insulin responses to an oral glucose challenge were reduced by 15% and 20%
DiPietro et al. [60]	1998	16 (7 of which serving as controls)	73 ± 1 y	Male and female	Moderate-intensity aerobic training, four times a week for 60-min sessions	Improvement in insulin resistance and glucose tolerance
Chadan et al. [54]	1999	7	62–69 y	Female	Four bouts of physical activity on separate occasions at either a low (heart rate = 100 bpm) or moderate intensity (heart rate = 120 bpm) for either 25 or 50 min	Decrease by 35% in all experimental conditions
Evans et al. [35]	2005	10	80.3 ± 2.5 y, 77–87 y	Male (n = 8) and female (n = 2)	10–12 months of program (for a total of 108 exercise sessions) consisting in a supervised endurance exercise training comprising of 2.5 sessions/week, 58 min/session, at an intensity of 83% of peak heart rate	Improvement in insulin activity
Goulet et al. [36]	2005	8 versus 14 younger controls	62.3 ± 4.7 y	Female	Aerobic training (25–60 min sessions of running at 60–95% of maximal heart rate) three days per week during 6 months, with insulin resistance measured 3–5 days after the last training bout	No improvement in insulin resistance
DiPietro et al. [66]	2006	25	73 ± 10 y	Female	Random allocation to high-intensity [80% peak aerobic capacity (VO_2peak_)] aerobic training, moderate-intensity (65% VO_2peak_) aerobic training, and low-intensity (stretching) placebo control (50% VO_2peak_) groups	Significant improvements only in the high-intensity training group
Bassami et al. [76]	2007	13	60 y and older	Male	Three 30-min trials on a cycle ergometer at 50%, 60% and 70% VO_2max_ and two other trials at 60% and 70% VO_2max_ in which the total energy expenditure was equal to that for 30 min at 50% VO_2max_	No significant differences between groups
Fujita et al. [55]	2007	13	70 ± 2 y	Male (n = 10) and female (n = 3)	Bout of aerobic exercise (45-min treadmill walk, 70% heart rate max)	Improvement in insulin resistance
Kodama et al. [56]	2007	56	64 ± 6 y	Male (n = 14) and female (n = 42)	Low-intensity and low-volume exercise training (12-week exercise program, comprising of aerobic training and resistance training)	Decrease in insulin resistance by 21%
Dipietro et al. [37]	2008	20	74 ± 5 y	Female	Random allocation into a high-volume, moderate-intensity aerobic (n = 12) and a lower-intensity resistance training (n = 8) groups 4 times per week for 45 to 60-min sessions over nine months	Not statistically significant changes in insulin levels in both groups
Dela et al. [72]	2011	42 (20 of which serving as controls)	60 y and older	Male and female	12 weeks of alpine ski training	Decrease in insulin concentration, decreased insulin resistance
Lira et al. [57]	2011	14	70.32 ± 0.72 y	Male	Moderate training for 60 min/d, 3 day/w for 24 weeks at a work rate equivalent to the ventilatory aerobic threshold	Improvement in insulin concentration and insulin resistance
Mikkelsen et al. [64]	2013	27 versus 22 young controls	NR	Male	Self-reported physical activity (n = 15 trained, n = 12 untrained)	Better insulin profile in trained subjects
Gando et al. [59]	2014	807	58-59 y	Male and female	Physical activity was measured using a triaxial accelerometer worn for 28 days and summarized as light intensity (1.1–2.9 METs) or moderate to vigorous intensity (≥ 3.0 METs)	Light physical activity inversely associated with insulin resistance
Hwang et al. [67]	2016	51 (16 of which serving as controls)	65 ± 1 y [55-79 y]	Male and female	Randomly allocated to high-intensity interval training (n = 17) or to moderate intensity continuous training (n = 18)	Insulin resistance decreased by 26% only in the high-intensity interval training group
Chen et al. [68]	2017	26	60–76 y	Male	Randomly allocated to the eccentric training or concentric training group (n = 13 per group), performing 30–60 eccentric or concentric contractions of knee extensors once a week. The intensity of the training program was progressively increased over a period of 12-weeks from 10% to 100% of maximal concentric strength for eccentric training and from 50% to 100% for the concentric training program	Statistically significant improvement of insulin sensitivity only after eccentric training
Herbert et al. [23]	2017	22 (cases) versus 17 (controls)	62 ± 2 y	Male	6 weeks of high-intensity interval training	Moderate reduction in insulin levels
Robinson et al. [73]	2017	26	60 y and older	Male (53.8%)	12 weeks of high-intensity aerobic interval, resistance, and combined exercise training	Increased insulin activity and sensitivity, with effects more marked in the high-intensity aerobic interval group
Banitalebi et al. [74]	2018	40 (12 of which serving as controls)	67.35 ± 1.40 y	Female	Randomly allocated to a resistance followed by endurance training program (n = 12), endurance training followed by resistance training (n = 12), interval resistance-endurance training (n = 12)	No differences among the groups and no difference between before and after the intervention
Lithgow and Leggate [53]	2018	14	64 ± 2 y	Male and female	Single bout of high intensity intermittent exercise	Insulin concentration during an OGTT elevated at 60 min when compared to the control trial
McGregor et al. [58]	2018	1,454	65–79 y	Male and female	Light-intensity physical activity and moderate to vigorous intensity physical activity assessed during the Canadian Health Measures Survey	2,000 steps/d can be sufficient to preserve insulin activity and sensitivity
Park et al. [65]	2018	2,325	60–74 y	Male (n = 862) and female (n = 1,463)	Self-reported physical activity level	OR of developing insulin resistance 0.55 [95%CI 0.34–0.87] in men and 0.68 [95%CI 0.47–0.98] in women
Søgaard et al. [77]	2018	22	63 ± 1 y	Male (n = 11) and female (n = 11)	High-intensity interval training three times/w for 6 weeks on a bicycle ergometer	Statistically significant improved insulin sensitivity
Ihalainen et al. [38]	2019	92 randomly assigned to a group performing strength training one-, two-, or three-times-per-w and a non-training control group	65–75 y	Male and female	Whole-body strength training using 2–5 sets and 4–12 repetitions per exercise and 7–9 exercises per session for 6 mo	No differences between groups and between before and after the intervention

Abbreviations: AUC (area under the curve); CI (confidence interval); d (day); MET (metabolic equivalent task); min (minute); mo (month); NR (not reported); OGTT (oral glucose tolerance test); OR (odds-ratio); w (week); y (years).

**Table 3:** Studies investigating the effects of physical activity and exercise on IGF-1 in elderly subjects.

Authors	Study Year	Sample Size	Age	Gender	Intervention	Main findings
Hagberg et al. [82]	1985	10 (cases versus 11 young trained subjects, 13 young sedentary subjects and 11 old trained subjects)	60–70 y	Male	Progressive VO_2max_ test and modified Balke protocol	No changes
Poehlman and Copeland [83]	1990	26 (cases versus 42 young controls)	59–76	Male	Self-reported physical activity level	IGF-1 level correlating with leisure time physical activity (r = 0.45; *p* < 0.01)
Poehlman et al. [84]	1994	18	66.1 ± 1.4 y	Male (n = 10) and female (n = 8)	8 weeks of endurance training	Increase in IGF-1 level by 14%
Vitiello et al. [85]	1997	67	60 y and older	Male (n = 46) and female (n = 21)	Randomized allocation to 3 d/w, 6-months endurance, stretching/flexibility groups and to 5-d/w, 6-months endurance protocol	No differences among the different experimental groups and between before and after the exercise interventions
Bermon et al. [86]	1999	32	67–80 y	Male (n = 16) and female (n = 16)	Randomly allocated to habitual physical activity or to an 8-week strength training program	Increase in total and free IGF-1 levels immediately after exercise (by 17.7% and 93.8%) and at 6 hours after exercise (by 7.5% and 31.2%)
Bonnefoy et al. [87]	1999	39	66–84 y	Male (n = 14) and female (n = 25)	Acute and chronic exercise (in a period of 6 months) evaluated using a self-administered questionnaire	IGF-1 levels correlated with sports activity
Chadan et al. [54]	1999	7	62–69 y	Female	Four bouts of physical activity on separate occasions at either a low (heart rate = 100 bpm) or moderate intensity (heart rate = 120 bpm) for either 25 or 50 min	No differences among the different experimental conditions
Ravaglia et al. [88]	2001	48	60 y and older	Male	Self-reported physical activity: active (n = 24) and inactive (n = 24)	Higher IGF-1 levels in active men
Borst et al. [89]	2002	62	68.1 y	Male and female	Randomly allocated to 6-month, 3-d/w program of low-intensity or high-intensity resistance training programs	No changes
Dennis et al. [90]	2008	16 versus 15 young controls	72 ± 5 y	Male	Acute resistance exercise	Higher levels of IGF-1 and IGFBP5 in younger subjects, especially after acute resistance exercise
Tsai et al. [91]	2015	48 (24 of which serving as controls)	71.40 ± 3.79 y (65–79 y)	Male	Long-term resistance exercise	Increase in IGF-1 levels
Maass et al. [92]	2016	40	60–77	Male	Pseudo-random allocation to aerobic exercise group (indoor treadmill, n = 21) or to a control group (indoor progressive-muscle relaxation/stretching, n = 19)	No changes
De Gonzalo-Calvo et al. [93]	2012	26 (active, n = 13, inactive, n = 13)	65 y and older	Male	49 ± 8 y of long-life training	Increase in IGF-1 concentration correlating with physical activity
Arnarson et al. [81]	2015	235	73.7 ± 5.7 y	Male (41.8%) and female (58.2%)	12-week resistance exercise program (3 times/w; 3 sets, 6–8 repetitions at 75–80% of the 1-repetition maximum)	Decrease in IGF-1 levels
Herbert et al. [23]	2017	22 (cases) versus 17 (controls)	62 ± 2 y	Male	12 weeks of preconditioning and 6 weeks of high-intensity training	Increase compared to baseline, and compared to preconditioning Preconditioning accounted for 8% of the increase from baseline
Negaresh et al. [94]	2017	15 versus 16 younger controls	60 y and older	Male	8 weeks of resistance training	No change in IGF-1 levels after training
Yoon et al. [95]	2017	21	65–75 y	Female	Randomly allocated to a low-intensity resistance training with heating sheet group (n = 8), a moderate-intensity resistance training (n = 6), and a heating sheet group (n = 7), over 12 weeks	Increased IGF-1 level
Banitalebi et al. [74]	2018	40	67.35 ± 1.40 y	Female	Randomized allocation to a resistance followed by endurance training (n = 12), endurance training followed by resistance training (n = 12, interval resistance-endurance training (n = 12) and a control (n = 12) groups	No differences among the groups and no difference between before and after the intervention
Cunha et al. [96]	2018	62 (21 of which serving as controls)	60 y and older	Female	Randomized allocation to a single set resistance training (n = 21) or multiple set resistance training (n = 20) programs, for 12 weeks using 8 exercises of 10–15 repetitions maximum for each exercise	Increase in IGF-1 levels (by 7.1% in the single set resistance training group and by 10.1% in the multiple set resistance training group)
Negaresh et al. [97]	2019	15	55–70 y	Male	Whole-body progressive resistance training program 3 d/w for 8 weeks (24 sessions)	Increase in IGF-1 levels

Abbreviations: d (day); mo (month); w (week); y (years).

**Table 4:** Studies investigating the effects of physical activities and exercise on growth hormone in elderly subjects.

Authors	Study Year	Sample Size	Age	Gender	Intervention	Main findings
Pyka et al. [107]	1992	11 versus 12 younger controls	72 ± 0.8 y	Male (n = 6) and female (n = 5)	3 sets of 8 repetitions for each of the 12 exercises, at 70% of 1RM values	Growth hormone response to resistance exercise abolished/diminished in elderly subjects
Cearlock and Nuzzo [108]	2001	9 versus 16 younger controls	60–85 y	Female	4-week exercise program followed by 1 w of no exercise	No changes
Banitalebi et al. [74]	2018	40 (12 of which serving as controls)	67.35 ± 1.40 y	Female	Randomized allocation to a resistance followed by endurance training program (n = 12), endurance training followed by resistance training (n = 12), interval resistance-endurance training (n = 12) groups	No changes

Abbreviations: RM (repetition maximum); w (week); y (years).
